# Efficacy and safety of obinutuzumab in primary membranous nephropathy: a real-world retrospective study

**DOI:** 10.3389/fimmu.2025.1650054

**Published:** 2025-08-21

**Authors:** Xi Cheng, Zhen-feng Zheng, Yan Qi, Xin Cao, Xi-qian Gao, Ying-xin Fang, Li Wei, Wen-ya Shang, Tie-kun Yan, Jun-ya Jia, Peng-cheng Xu, Qiu-hua Gu

**Affiliations:** ^1^ Department of Nephrology, Kidney Disease Medical Center, Tianjin Medical University General Hospital, National Key Clinical Specialty, Tianjin Key Medical Discipline, Tianjin, China; ^2^ Department of Nephrology, Tianjin Medical University General Hospital Airport Hospital, Tianjin, China

**Keywords:** membranous nephropathy, obinutuzumab, rituximab, anti-phospholipase A2 receptor antibody, renal function

## Abstract

**Objective:**

This study aimed to evaluate the efficacy and safety of obinutuzumab, a novel anti-CD20 monoclonal antibody, in patients with primary membranous nephropathy (pMN).

**Methods:**

Fifty-five patients with pMN treated with obinutuzumab were respectively enrolled in this study. Clinical and immunological response, renal function and adverse events were assessed throughout the follow-up period between patients receiving obinutuzumab as initial therapy and alternative therapy.

**Results:**

The study included 40 patients receiving obinutuzumab as an alternative therapy, and 15 patients receiving it as an initial therapy. During a follow up of 13.0(10.0, 18.0) months after obinutuzumab treatment, 23/25(92.0%) patients achieved immunological remission. 46/55(83.6%) patients achieved clinical remission (including 40.0% of PR and 43.6% of CR), with significantly reduced proteinuria and increased serum albumin. Patients with obinutuzumab as either the initial or alternative treatment showed similar clinical response (85.0 vs. 80.0%, *P*=0.692). 13 out of 18 patients (72.2%) with impaired kidney function (eGFR <60 mL/min/1.73 m²) also showed significant response to obinutuzumab, along with eGFR increasing from 35.3 to 47.6 mL/min/1.73m². Furthermore, obinutuzumab also had a comparable efficiency in patients without response to previous rituximab treatment and patients with negative anti-PLA2R antibody. More patients in the initial therapy group had infusion-related reactions (33.3 vs. 15.0%, *P*=0.149), while severe infections were all occurred in the alternative therapy group, particularly those with a history of long-term immunosuppressive therapy.

**Conclusions:**

Obinutuzumab, used as both initial and alternative therapy, can induce significant clinical response in patients with pMN, implying its potentially promising therapeutic effect on pMN.

## Introduction

Membranous nephropathy (MN) is one of the most common causes of nephrotic syndrome in adults, characterized by the accumulation of immune complexes in the sub-epithelial layer of the glomerular capillary wall, which is composed of immunoglobulins and complement components. As an autoimmune kidney disease, several autoantigens in podocytes have been identified, with M-type phospholipase A2 receptor (PLA2R) as the main target antigen (1). Additionally, circulating auto-antibody against PLA2R could be detected in approximately 70% of patients with primary MN (pMN) (1) and has predictive value for the clinical remission and disease flare in patients with pMN (2, 3), furtherly supporting the key role of B cells in the pathogenesis and development of pMN.

B-cell targeted therapy with rituximab, a type I anti-CD20 antibody, has been demonstrated as an effective treatment for about 60-80% patients with MN and showed unique superiority to the traditional therapy, including prednisone combined with cyclophosphamide and calcineurin inhibitors (4–6), leading to its recommendation as the first-line therapy for pMN by the KDIGO guidelines (7). However, the real-world studies reported that about 20-40% patients with pMN were resistant to rituximab (8–11). The resistance might be attributed to the production of anti-rituximab antibody, internalization and destruction of rituximab or the urinary loss combined with other urinary protein (11), highlighting the need for exploration of novel treatment strategies. Obinutuzumab, a humanized de-fucosylated IgG1 monoclonal antibody against CD20, targets a different epitope on CD20 from rituximab (12). Though obinutuzumab induces a markedly reduced complement-dependent cytotoxicity (CDC) effect, it has more potent induction of direct cell death (DCD) and more effective antibody-dependent cellular cytotoxicity (ADCC) than rituximab, resulting from its differing ability to redistribute CD20 molecules on the cell surface and its differing pattern of glycoengineered Fc fragment (13, 14). Based on all of that, obinutuzumab yields a profound ability to induce B cell apoptosis and CD20 depletion.

The latest studies have indicated that patients with either untreated or refractory MN exhibited a better response to obinutuzumab than rituximab (15, 16). Furthermore, several case reports and small-size studies in recent years have also demonstrated that, for 80-90% of patients who were refractory to traditional immunosuppressive therapy, including rituximab, obinutuzumab treatment could still induce clinical and immunological remission (16–21). Additionally, an observational case series also observed the promising potential of obinutuzumab as an initial treatment for pMN (22), which has been also highlighted in another retrospective study from a single center by comparison with patients using obinutuzumab as an alternative treatment (23). However, most of the studies are small-scale, and the safety and efficacy of obinutuzumab in real-world clinical settings also needs to be verified in different groups of patients with pMN, such as patients with impaired kidney function.

In this study, we conducted a retrospective analysis of patients with pMN who were treated with obinutuzumab as either alternative therapy or as initial therapy, aiming to evaluate the efficacy and safety of obinutuzumab in real-world clinical practice, and to provide additional evidence for the therapy and management of patients with different stage of MN.

## Materials and methods

### Patients

A total of 55 patients with pMN undergoing obinutuzumab treatment from September 2021 to December 2023 in Tianjin Medical University General Hospital were included in the study. All the patients fulfilled the following criteria: (i) patients with biopsy-proven MN, or lacking renal biopsy but with a high level of anti-PLA2R antibody (>50RU/ml) at baseline; (ii) patients without secondary factors, including autoimmune diseases (e.g., systemic lupus erythematosus and rheumatoid arthritis), infections (e.g., hepatitis B/C virus, syphilis and human immunodeficiency virus), malignancies, heavy metal poisoning and medications; (iii) patients with a follow-up period for more than 6 months. The research was approved by the ethics committee of Tianjin Medical University General Hospital. Informed consent was obtained for each patient.

### Obinutuzumab treatment and follow-up

Obinutuzumab therapy was initiated with an intravenous infusion of 1g, following premedication with 80mg of intravenous methylprednisolone to reduce the risk of infusion-related reactions. After the initial administration, repeated infusions of obinutuzumab were administrated at a dosage of 1g by single usage. The interval between the first and second infusions were typically 2 weeks. Participants were divided into the initial therapy group and the alternative therapy group according to whether they had previously received immunosuppressant or rituximab medication before starting obinutuzumab therapy.

Laboratory evaluations, including urinary protein/24h, serum albumin, creatinine, and anti-PLA2R antibody were performed at baseline and each visit. Lymphocyte subsets were assessed within one week before the initial obinutuzumab infusion and the day after the second infusion. Adverse events related to obinutuzumab were monitored during the drug infusion and throughout the follow-up period. The follow-up period for all patients was defined as commencing from the initiation of obinutuzumab therapy (the date of the first dose) and continuing until the date of their final visit during the study period. The clinical endpoint was defined as end stage kidney disease (ESKD) (eGFR<15 mL/min/1.73 m^2^ or depending on renal replacement therapy), eGFR decline ≥40%, or death.

### Treatment responses and renal outcomes

Clinical response was evaluated based on the established criteria (7). Complete remission was defined as urinary protein <0.3g/24h, with normal level of serum albumin and normal kidney function. Partial remission was defined as urinary protein <3.5g/24h with a 50% reduction from peak values, along with improvement or normalization of serum albumin level, and stable kidney function. Patients who did not meet the definitions above were considered to be non-responders. Relapse was defined as recurrence of proteinuria ≥3.5g/day following a period of partial or complete response. Serum anti-PLA2R antibody level >5RU/ml, as measured by ELISA were considered as positive. B-cell depletion was defined as number of CD19-positive cell fewer than 5 cells/μL (15).

### Renal histopathology

Renal biopsy was performed in 43/55(78.2%) patients, including 35 in our center and 8 in other hospitals. The renal biopsy specimens were assessed by light microscopy, direct immunofluorescence, and electron microscopy, as detailed in our previous study (24).

### Safety assessment

Safety assessments encompassed statistics on mortality, infusion-related reaction and adverse events (AEs), including infections and non-infections. AEs were systematically recorded during each clinical evaluation and monitored throughout the follow-up periods.

### Statistical analyses

Statistical software SPSS version 25.0 (SPSS, Chicago, IL, USA) was utilized for statistical analysis. Categorical data were reported as counts and percentages. Differences in qualitative data were compared using either the chi-squared test or Fisher’s exact test. Quantitative variables between two groups that were normally distributed were expressed as mean ± standard deviation (SD) and compared by student’s t test, while non-normally distributed quantitative variables were expressed as median with interquartile range and compared by Wilcoxon test. Kaplan-Meier curve was used to plot the probability of achieving clinical response, with Log-rank test applied to analyze differences between groups. Cox regression analyses were performed to confirm potential risk or protective factors for treatment responses. All statistical analyses were two-tailed and *P* value <0.05 was considered as statistically significant.

## Results

### Clinical and pathological features of patients

**Figure 1:**
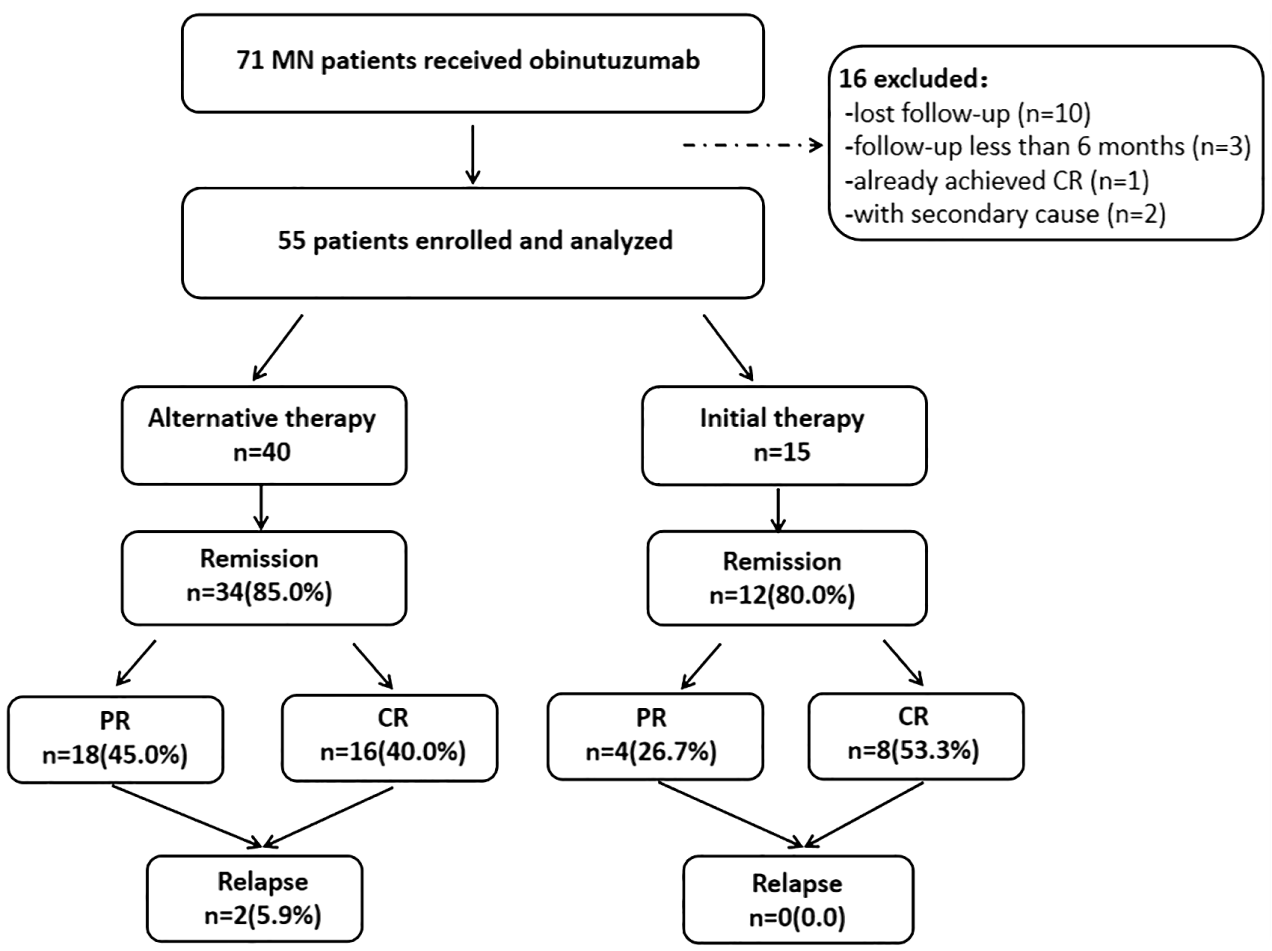
Flowchart of the study. MN, membranous nephropathy; PR, partial remission; CR, complete remission.

A total of fifty-five patients diagnosed with pMN and treated with obinutuzumab were enrolled and analyzed in the current study (**Figure 1**). The cohort consisted of 34 male and 21 female patients, with a mean age of 54.7 ± 14.1 years. At the time of enrollment, the median level of proteinuria was 5.4(3.7, 10.9) g/24h, with the median levels of serum albumin as 23.0(19.0, 30.0) g/L, and eGFR as 76.3(51.6, 108.0) mL/min/1.73m^2^. Notably, 18 out of 55(32.7%) patients had a decreased kidney function, with eGFR <60mL/min/1.73 m^2^. Among the 39(70.9%) patients with detectable circulating anti-PLA2R antibody, the median level of anti-PLA2R antibody was 22.7(5.0, 108.0) RU/mL. (**Table 1**).

**Table 1:** Baseline characteristics of patients with pMN included in this study.

Parameter	Total (N=55)	Alternative therapy (N=40)	Initial therapy (N=15)	*P-* value
Gender (M/F), n	34/21	28/12	6/9	0.062
Age (years)	54.7 ± 14.1	55.2 ± 13.3	53.5 ± 16.4	0.123
Diabetes mellitus, n (%)	15(27.3)	12(30.0)	3(20.0)	0.458
Hypertension, n (%)	29(52.7)	24(60.0)	5(33.3)	0.078
Serum albumin (g/L)	23.0(19.0, 30.0)	26.0(22.0, 31.0)	20.0(17.0, 23.0)	**0.009**
Urinary protein (g/24h)	5.4(3.7, 10.9)	5.0(3.6, 10.3)	7.9(3.7, 13.1)	0.245
Serum creatinine (μmol/L)	82.0(61.0, 127.0)	92.0(61.3, 136.0)	70.0(42.0, 96.0)	0.060
eGFR (mL/min/1.73 m^2^)	76.3(51.6, 108.0)	66.1(44.3, 107.3)	79.0(73.7, 125.3)	0.146
eGFR < 60mL/min/1.73 m^2^, n (%)	18(32.7)	16(40.0)	2(13.3)	0.105
Level of anti-PLA2R antibody (RU/mL)	22.7(5.0, 108.0)	9.3(5.0, 51.0)	101.4(18.3, 402.8)	**0.015**
Positivity of anti-PLA2R antibody, n (%)	39(70.9)	26(65.0)	13(86.7)	0.115
B cell counts (cells/μL)	164.5(35.3, 244.5)	109.0(34.3, 266.0)	172.5(170.0, 194.6)	0.602
T cell counts (cells/μL)	1603.0(1206.8, 1826.8)	1518.0(1174.5, 1939.0)	1749.0(1666.0, 1835.5)	0.465
NK cell counts (cells/μL)	291.0(186.5, 468.5)	279.0(182.0, 474.8)	394.0(316.0, 422.6)	0.403

MN, membranous nephropathy; eGFR, estimated glomerular filtration rate; PLA2R, M-type phospholipase A2 receptor.

Values in bold indicate statistically significant differences (p < 0.05).

In our cohort, 40 patients received obinutuzumab as an alternative therapy, while 15 patients received it as an initial therapy. The demographic and clinical characteristics of patients in these two groups at the beginning of obinutuzumab treatment were summarized in **Table 1**. Among the 40 patients in the alternative therapy group, 39(97.5%) patients were anti-PLA2R antibody positive at the time of diagnosis with a median level of 99.1(39.8, 402.5) RU/mL (**Supplementary Table S1**). At the diagnosis of pMN, all of them showed nephrotic syndrome, with a high level of urinary protein 6.5(3.9, 12.0) g/24h and a low level of serum albumin 22.0(16.0, 29.0) g/L, as well as almost normal kidney function, with serum creatinine of 73.0(60.0, 84.0) μmol/L and eGFR of 92.9(81.3, 107.2) mL/min/1.73m^2^, respectively (**Supplementary Table S1**). All patients in the alternative therapy group had been treated with at least one round of immunomed therapy prior to obinutuzumab treatment, with 57.5% patients having received at least two rounds of immunosuppressive treatment (**Supplementary Table S1**). The immunosuppressants previously administered to patients in the alternative therapy group included rituximab in 30(75.0%) patients, cyclophosphamide in 9(22.5%) patients, calcineurin inhibitor in 23(57.5%) patients, and 10(25.0%) patients previously using other immunosuppressant, including tripterygium wilfordii, leflunomide and mycophenolate mofetil (MMF) (**Supplementary Table S1**). Among these 40 patients, prior to receiving obinutuzumab, 17 (42.5%) had been continuously treated with the previous immunosuppressive regimen for 8(3.5, 16.0) months. The remaining 23 patients had discontinued rituximab or other immunosuppressive therapies for 8.0(6.0, 12.0) months. Only six of the forty patients achieved complete or partial remission during prior therapies. The remaining 34(85%) patients did not respond to any regimen (**Supplementary Table S1**).

At the initiation of obinutuzumab treatment, compared to the patients in the initial therapy group, patients in the alternative therapy group presented with a numerically higher proportion of eGFR <60mL/min/1.73 m^2^ (40.0 vs. 13.3%, *P*=0.105). However, for the patients with obinutuzumab as the alternative therapy, the level of serum albumin was significantly higher (26.0, 22.0-31.0 vs. 20.0, 17.0-23.0g/L, *P*=0.009) and the level of anti-PLA2R antibody was significantly lower (9.3, 5.0-51.0 vs. 101.4, 18.3-402.8RU/mL, *P* =0.015), though the median levels of urinary protein, serum creatinine and eGFR were comparable between these two groups. In addition, the circulating B cell, T cell and NK cell counts were comparable between these two groups at the administration of obinutuzumab treatment (*P*>0.05). (**Table 1**).

Overall, 43/55 (78.2%) patients underwent renal biopsy identifying the diagnosis of MN, including 32 patients in the alternative therapy group and 11 patients in the initial therapy group. And the other 12 patients exhibited a high level of circulating anti-PLA2R antibody, confirming the diagnosis of MN, though without kidney pathological assessment. According to the histopathological manifestations, Churg stages showed no significant difference between patients in the initial and alternative therapy group. IgG (predominantly IgG1 and IgG4) and C3 deposits were observed in all patients. Additionally, granular deposits of IgA, IgM and C1q were present in some patients, with no significant difference between patients in the initial and alternative therapy group. Positive PLA2R staining in the glomeruli was observed in 12/12(100.0%) patients in the alternative therapy group and in 81.8% (9/11) of those in the initial therapy group (*P*=0.217). (**Supplementary Table S2**).

### Immunological responses

**Figure 2:**
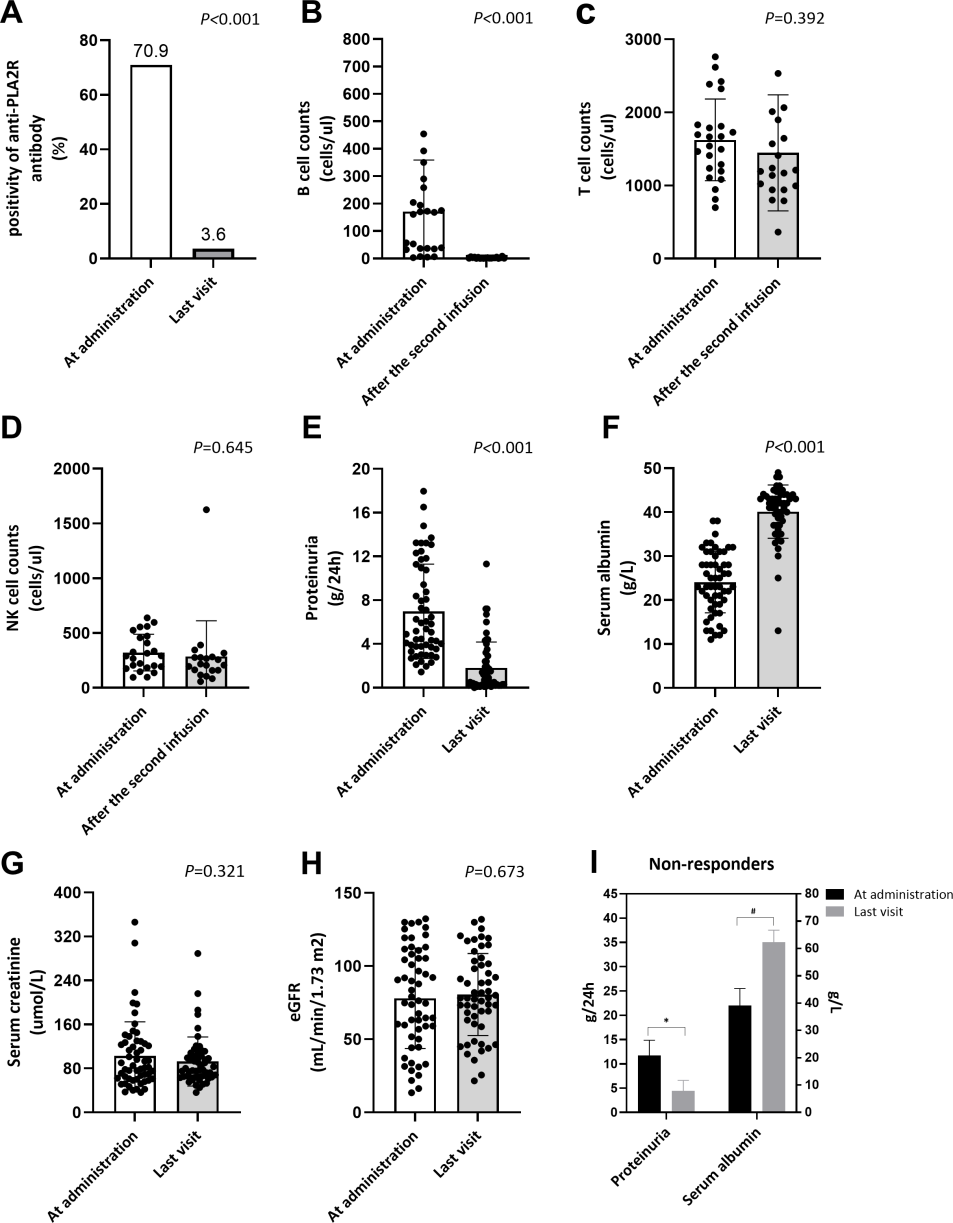
Renal response and lymphocyte subsets in patients with pMN after obinutuzumab treatment. Comparison of **(A)** positivity of anti-PLA2R antibody between the time of obinutuzumab administration and the last visit of follow-up; Changes of **(B)** B cell counts, **(C)** T cell counts and **(D)** NK cell counts from the first obinutuzumab infusion to the day after the second infusion; Comparison of proteinuria **(E)**, serum albumin **(F)**, serum creatinine **(G)** and eGFR **(H)** between the time of obinutuzumab administration and the last visit of follow-up; **(I)** Proteinuria and serum albumin of non-responders at administration of obinutuzumab and at the last visit of follow-up; eGFR: estimated glomerular filtration rate; ^*^ *P* < 0.05; ^#^ *P* < 0.05.

After obinutuzumab treatment, 23/25(92.0%) patients got immunological remission with undetectable anti-PLA2R antibody in the circulation, during follow-up of 13.0(10.0, 18.0) months (**Table 2**). Overall, the rate of anti-PLA2R antibody positivity declined from 70.9% at the administration of obinutuzumab to 3.6% at the last visit (**Figure 2A**). Circulating CD19^+^ B-cell counts decreased from 164.5(35.3, 244.5) to 2.0(1.0, 3.8)cells/μL (*P*<0.001) within 2 weeks after obinutuzumab infusion (**Table 2**, **Figure 2B**). However, the number of T cells (1603.0, 1206.8-1826.8 to 1202.0, 955.5-1835.0cells/μL, *P*=0.392) and NK cells (291.0, 186.5-468.5 to 212.5, 159.3-281.0cells/μL, *P*=0.645) remained stable after B cell clearance (**Table 2**, **Figures 2C, D**).

**Table 2:** Treatment response of obinutuzumab in patients with pMN.

Parameter	Total (N=55)	Alternative therapy (N=40)	Initial therapy (N=15)	*P-* value
Follow-up duration (months)	13.0(10.0, 18.0)	13.0(10.0, 18.8)	12.0(9.0, 16.0)	0.161
At 2 weeks				
B cell counts (cells/μL)	2.0(1.0, 3.8)	2.0(1.0, 3.0)	2.0(0.5, 5.0)	0.847
T cell counts (cells/μL)	1202.0(955.5, 1835.0)	1411.0(1172.0, 2010.0)	996.0(870.5, 1428.5)	0.102
NK cell counts (cells/μL)	212.5(159.3, 281.0)	267.0(159.0, 346.0)	207.0(135.0, 267.0)	0.342
At last visit				
Serum albumin (g/L)	42.0(37.0, 44.0)	42.0(39.0, 44.0)	41.0(33.0, 43.6)	0.178
Proteinuria (g/24h)	0.8(0.2, 2.4)	0.9(0.3, 2.3)	0.3(0.2, 4.3)	0.734
Serum creatinine (μmol/L)	78.5(64.8, 106.5)	90.0(66.0, 111.0)	72.0(55.0,95.0)	**0.026**
eGFR (mL/min/1.73 m^2^)	78.3(62.4, 102.0)	78.2(48.6, 100.5)	80.0(73.2, 119.1)	0.251
eGFR < 60mL/min/1.73 m^2^, n (%)	12/54(22.2)	11/39(28.2)	1(6.7)	0.088
Immunological remission	23/25(92.0)	15/16(93.8)	8/9(88.9)	0.667
Clinical response, n (%)	46(83.6)	34(85.0)	12(80.0)	0.692
Partial remission, n (%)	22(40.0)	18(45.0)	4(26.7)	0.354
Complete remission, n (%)	24(43.6)	16(40.0)	8(53.3)	0.543
Relapse, n (%)	2/46(4.3)	2/34(5.9)	0(0)	1.000
Time to remission (months)	9.0(7.0, 13.0)	9.0(6.0, 13.0)	12.0(8.0, 16.0)	0.115

Values in bold indicate statistically significant differences (p < 0.05).

### Clinical outcomes

Briefly, after obinutuzumab treatment, urinary protein significantly decreased from 5.4(3.7, 10.9) to 0.8(0.2, 2.4) g/24h and serum albumin significantly increased from 23.0(19.0, 30.0) to 42.0(37.0, 44.0) g/L (*P*<0.001, **Table 2**, **Figures 2E, F**). Additionally, all the patients maintained a stable kidney function during follow-up, with eGFR of 76.3(51.6, 108.0) mL/min/1.73 m^2^ before obinutuzumab treatment and 78.3(62.4, 102.0) mL/min/1.73 m^2^ at the end of follow-up (*P*=0.673, **Table 2**, **Figures 2G, H**).

**Figure 3:**
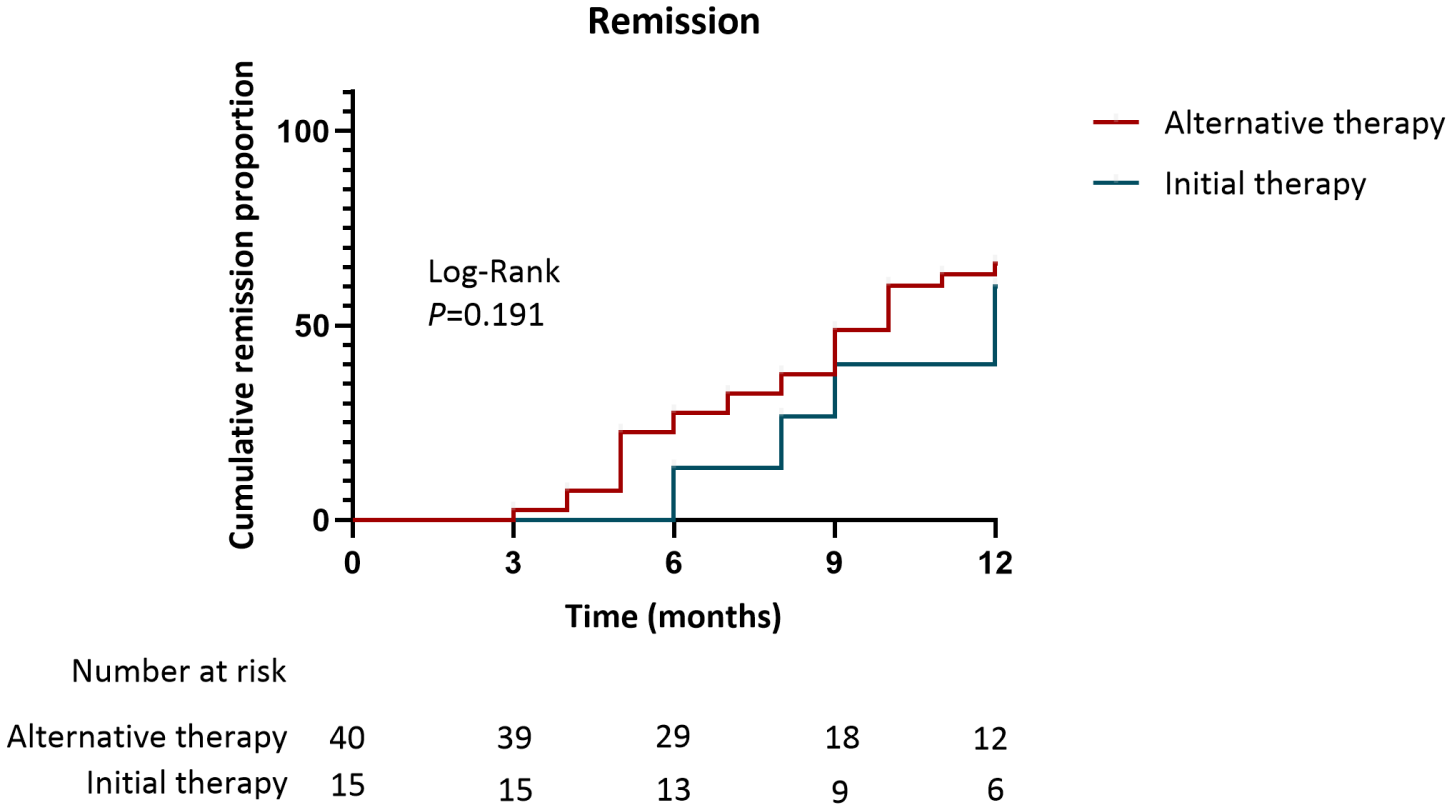
Kaplan-Meier curves for the cumulative incidence of clinical remission in patients received obinutuzumab as alternative therapy and initial therapy.

During a period of 13.0(10.0, 18.0) months, 34(85.0%) patients with obinutuzumab therapy as their alternative treatment achieved remission, including CR of 16(40.0%) patients and PR of 18(45.0%) patients, which were comparable with patients receiving obinutuzumab therapy as initial treatment (85.0 vs. 80.0%, *P*=0.692) (**Table 2**, **Figure 1**). The median time for achieving clinical response was also comparable between these two groups (9.0, 6.0-13.0 vs. 12.0, 8.0-16.0months, *P*=0.115) (**Table 2**). The cumulative incidence of remission showed no difference when obinutuzumab was treated as either initial therapy or alternative therapy (Log-Rank, *P*=0.191, **Figure 3**). However, following treatment, kidney function in the alternative therapy group remained worse than that in the initial therapy group, consistent with differences observed at baseline. Furthermore, there were no differences in immunological remission and post-treatment levels of serum albumin and proteinuria between patients with obinutuzumab as either initial therapy or alternative therapy (**Table 2**).

After the treatment of obinutuzumab, though 46 patients reached clinical response, there were still 9 patients showed no response to treatment (**Table 3**). Compared to the responders, patients with no response showed numerically higher baseline level of urinary protein (11.7, 4.2-14.9 vs. 5.1, 3.6-8.5g/24h, *P*=0.069) and significantly higher baseline level of anti-PLA2R antibody (125.3, 71.6-248.9 vs. 11.5, 5.0-56.2RU/mL, *P*=0.006), as well as a significant lower level of eGFR (59.0, 31.2-78.7 vs. 85.7, 58.5-111.9mL/min/1.73m^2^, *P*=0.007) (**Table 3**). Moreover, the proportion of patients with eGFR <60mL/min/1.73m^2^ was significantly higher in the non-responder group compared to the responder group (77.8 vs. 23.9%, *P*=0.004). Among treatment responders, 30 of 46 patients tested positive for anti-PLA2R antibody, representing a significantly lower positivity rate than that observed in non-responders (65.2 vs. 100%, *P*=0.046). Other clinical characteristics didn’t show any significant differences in responders and non-responders at initial treatment of obinutuzumab (**Table 3**). Additionally, for the non-responders, though no significant remission were observed, obinutuzumab therapy significantly decreased the level of urinary protein and improved the level of serum albumin (**Figure 2I**). Univariate Cox regression analysis also indicated that the level of urinary protein (HR=0.916, 95%CI= 0.848-0.990, *P*=0.027) and serum albumin (HR=1.047, 95%CI=1.003-1.092, *P*=0.036), accompanied with eGFR<60mL/min/1.73m^2^ (HR=0.448, 95%CI=0.220-0.991, *P*=0.027) were affecting factors for renal remission after obinutuzumab treatment (**Table 4**). However, multivariate Cox regression analysis only identified eGFR<60mL/min/1.73m^2^ (HR=0.374, 95%CI=0.152-0.922, *P*=0.033) as the independent risk factor for response to obinutuzumab treatment (**Table 4**).

**Table 3:** Clinical characteristics in responders and non-responders at the initial treatment of obinutuzumab.

Parameter	Responders (N=46)	Non-responders (N=9)	*P*-value
Gender (M/F), n	30/16	4/5	0.279
Age (years)	54.1 ± 14.1	58.0 ± 14.4	0.448
Follow-up duration (months)	13.0(10.0, 18.3)	13(8.0, 17.0)	0.758
Rounds of previous immunomeds	2.0(1.0, 3.0)	2.0(1.0, 6.0)	0.524
Proteinuria (g/24h)	5.1(3.6, 8.5)	11.7(4.2, 14.9)	0.069
Serum albumin (g/L)	25.0(19.0, 31.0)	22.0(17.0, 25.5)	0.289
eGFR (mL/min/1.73 m^2^)	85.7(58.5, 111.9)	59.0(31.2, 78.7)	**0.007**
eGFR < 60mL/min/1.73 m^2^, n (%)	11(23.9)	7(77.8)	**0.004**
Anti-PLA2R antibody (RU/mL)	11.5(5.0, 56.2)	125.3(71.6, 248.9)	**0.006**
Positivity of anti-PLA2R antibody, n (%)	30/46(65.2)	9(100.0)	**0.046**
B cell counts (cells/μl)	161.9 ± 193.5	187.0(69.5, 391.5)	0.353
Total dose of obinutuzumab	2.0(2.0, 3.0)	2.0(2.0, 3.0)	0.842

MN, membranous nephropathy; eGFR, estimated glomerular filtration rate; PLA2R, M-type Phospholipase A2 receptor.

Values in bold indicate statistically significant differences (p < 0.05).

**Table 4:** Cox regression analysis of risk factors affecting renal remission after treatment of obinutuzumab.

Parameters	Univariate Cox regression analysis			Multivariate Cox regression analysis		
	HR	95%CI	*P*-value	HR	95%CI	*P*-value
Gender(male)	1.359	0.719-2.567	0.345	0.849	0.387-1.863	0.683
Age (years)	1.000	0.980-1.021	0.991	1.006	0.983-1.030	0.608
Rounds of previous immunomeds	0.898	0.689-1.171	0.429			
Proteinuria (g/24h)	0.916	0.848-0.990	**0.027**	0.979	0.884-1.083	0.675
Serum albumin (g/L)	1.047	1.003-1.092	**0.036**	1.045	0.988-1.105	0.123
eGFR (mL/min/1.73 m^2^)	1.006	0.997-1.014	0.179			
eGFR < 60mL/min/1.73 m^2^(Yes)	0.448	0.220-0.911	**0.027**	0.374	0.152-0.922	**0.033**
Anti-PLA2R antibody (RU/mL)	1.000	0.999-1.001	0.514			
Positivity of anti-PLA2R antibody	1.275	0.691-2.352	0.436			
B cell counts (cells/μl)	0.999	0.997-1.001	0.337			
Total dose of obinutuzumab	0.939	0.665-1.325	0.719			

Values in bold indicate statistically significant differences (p < 0.05).

In the alternative group, 30 patients received treatment of rituximab previously, among which 14/30(46.7%) patients achieved CR and 12/30(40.0%) patients achieved PR successfully, which were comparable with those who hadn’t received previous rituximab treatment (including 6/10 PR and 2/10 CR) (**Supplementary Table S3**). The median time to remission (either PR or CR) after the initial dose of obinutuzumab between patients with or without rituximab treatment was also similar (10, 6.8-13.0 vs. 7.5, 5.0-10.0 months, *P*=0.209) ( **Supplementary Table S3**). As shown in the Kaplan–Meier curve, the likelihood of achieving proteinuria remission was comparable between patients who previously received rituximab and those who did not. (Log-Rank *P*=0.442, **Supplementary Figure S1**).

Among patients with positive anti-PLA2R antibody, 30 out of 39 (76.9%) achieved a clinical response, which was lower than the remission rate observed in anti-PLA2R antibody-negative patients (76.9 vs. 100.0%, *P*=0.046). However, the CR rates were comparable between these two groups, with 43.6% in the anti-PLA2R antibody-positive group and 43.8% in the negative group (*P*=1.000). (**Supplementary Table S4**).

Furthermore, eighteen patients in our cohort had a compromised kidney function with eGFR<60 mL/min/1.73 m^2^. Among them, 13(72.2%) patients achieved clinical response, including 6/18(33.3%) patients achieving CR and 7/18(38.9%) patients of PR. For the patients with eGFR<60 mL/min/1.73 m^2^ in our cohort, after obinutuzumab treatment, levels of urinary protein (4.1, 3.2-12.5 to 1.1, 0.2-4.1g/24h, *P*<0.01) and anti-PLA2R antibody (48.1, 5.0-138.1 to 5.0, 5.0-5.0RU/mL, *P*<0.001) significantly decreased, while levels of eGFR (35.3, 27.8-52.9 to 47.6, 43.3-69.2mL/min/1.73 m^2^, *P*<0.05) and serum albumin (24.0, 21.8-32.0 to 40.0, 37.0-42.0, *P*<0.001) significantly increased. ( **Supplementary Table S5**).

### Relapse

Among the 46 patients who achieved remission, only two patients experienced a relapse during follow-up, whereas the remaining patients maintained their remission status until the end of the follow-up. Both two patients showed no response to 4 rounds of immunomeds (including rituximab, cyclophosphamide, calcineurin inhibitor and mycophenolate mofetil). However, an alternative therapy of obinutuzumab successfully help them achieve partial remission, with a depletion of circulating B cell and complete immunological remission (anti-PLA2R antibody <5RU/ml). In addition, kidney function also showed great improvement, with serum creatinine decreased from 346 to 153μmol/L and 101 to 91μmol/L, respectively. However, both of them relapsed respectively 25 months and 11 months later after achieving PR.

### Associated AEs

Adverse events were observed shortly after obinutuzumab treatment and during follow-up. 35 adverse events were reported in 21 patients. The major adverse events in both groups were infusion-related reactions and infections. Infusion-related reactions, such as chill and fever, digestive symptoms, dyspnea and palpitation occurred in 11(20.0%) patients with 14 events. And patients receiving obinutuzumab as alternative therapy had a lower frequency of infusion-related reactions, compared to the patients with obinutuzumab as an initial treatment (15.0 vs. 33.3%, *P*=0.149). All the reactions related with infusion occurred at the first time they received the obinutuzumab infusion, and completely relieved by a temporary interruption of infusion or a slower injection speed.

Thirteen patients experienced a total of 16 events of infections, including upper respiratory tract, urinary tract, soft tissue and biliary tract infections, pneumonia, and herpes zoster, with no significant difference in patients receiving obinutuzumab as alternative or initial therapy (25.0 vs. 20.0%, P=1.000). Upper respiratory tract, skin soft tissue infections and herpes zoster were completely resolved after oral antibiotics outside the hospital. Five patients developed pneumonia caused by COVID-19, fungus or bacterial. And one of them experienced severe infection with different pathogens successively but was successfully recovered after hospitalization. The patients with biliary tract infection and urinary tract infection were found to have biliary tract stones and urinary tract stones, and their conditions were improved after hospitalization. It’s noted that severe infections requiring hospitalization all occurred in the alternative treatment group. As for the other AEs other than infection, only 5 events (including abnormal liver function, prosopoplegia, amenorrhea and tinnitus) occurred, with 3 events in the group of alternative therapy and 2 events in the initial therapy group (*P*=0.606). One patient in the alternative group died from respiratory failure associated with pneumonia during the study period. (**Table 5**).

**Table 5:** Adverse events in patients with pMN after obinutuzumab treatment.

Parameter	Total (N=55)	Alternative therapy (N=40)	Initial therapy (N=15)	*P*-value
Infusion-related reaction, n (%)	11 (20.0)	6 (15.0)	5 (33.3)	0.149
Chill and fever, n	8	5	3	
Digestive symptoms, n	1	0	1	
Dyspnea, n	3	1	2	
Palpitation, n	2	1	1	
Infections, n (%)	13 (23.6)	10 (25.0)	3 (20.0)	1.000
Upper respiratory tract infection, n	6	4	2	
Soft tissue infection, n	1	1	0	
Herpes zoster, n	2	1	1	
Pneumonia, n	5	5	0	
Urinary tract infection, n	1	1	0	
Biliary tract Infection, n	1	1	0	
Non-infections	5 (9.1)	3 (7.5)	2 (13.3)	0.606
Abnormal liver function, n	2	1	1	
Prosopoplegia, n	1	0	1	
Amenorrhea, n	1	1	0	
Tinnitus, n	1	1	0	
Deaths	1 (1.8)	1 (2.5)	0 (0)	1.000
Total No. of adverse events, n	35	23	12	
Patients with adverse events, n (%)	21 (38.2)	14 (35.0)	7 (46.7)	0.537

## Discussion

B cell deletion-based therapy has become more competitive in the treatment of patients with pMN for its efficacy and fewer adverse events. In addition to rituximab, several studies have demonstrated that obinutuzumab, the type II monoclonal antibody against CD20, could induce more significant immunological and clinical remission (15, 16). However, more studies are needed to elucidate the efficacy and safety of obinutuzumab in different subgroup of pMN. The current study provides more evidence for the usage of obinutuzumab in the management of pMN.

Though the initial attempt to use obinutuzumab to treat pMN was for patients who were refractory nephrotic syndrome, successful outcomes were also obtained in most patients. In this study, our results also found that 85.0% of patients with pMN who received obinutuzumab as an alternative therapy could achieve clinical remission, which is consistent to previous case series (17). However, Xu et al. found patients with MN refractory to GC+CTX and/or CNIs were even successfully treated with obinutuzumab, with a remission rate as high as 90% (15). Similarly, for the untreated patients with pMN, studies from Su et al. and Hu et al. reported that the clinical remission was as high as 90% and 95% respectively (16, 23), indicating a better response than the patients receiving obinutuzumab as an alternative therapy. However, the study from Hao et al (22) in 12 patients with pMN and our results found the rate of clinical remission for patient using obinutuzumab as initial treatment was a little lower (83.3% and 80.0%, respectively), which probably was due to the different cohorts in different centers. Nevertheless, the therapeutic effects of obinutuzumab, as both the alternative therapy and the initial therapy in patients with pMN, are confirmed. (**Supplementary Table S6**).

Eighteen patients with eGFR less than 60mL/min/1.73m^2^ were enrolled in our study. We analyzed the efficacy of obinutuzumab in patients with compromised kidney function by comparing the patients in our study and previous studies. In our cohort, 72.2% of patients with eGFR less than 60mL/min/1.73m^2^ obtained clinical remission, which was consistent with the studies from published case reports (72.2% and 66.7%). However, compared to previous studies, our cohort has a numerically higher rate of complete remission (33.3 vs 13.3%, *P*=0.242), which may result from the lower level of urinary protein at the time of administration. After treatment with obinutuzumab, both cohorts experienced significant reductions in proteinuria and anti-PLA2R antibody levels, along with a notable increase in serum albumin. Moreover, renal function also improved significantly in our cohort. In addition, in the cohort of references, eGFR also slightly increased, despite with no significant difference. These findings indicates that obinutuzumab can also significantly improve renal function and induce clinical remission in more than 60% of patients with moderate renal impairment. (**Supplementary Table S5**).

Considering the similar target to CD20 with rituximab, whether obinutuzumab could still have effect on patients who were resistant to rituximab is critical for the management of MN. In our cohort, twenty-six out of thirty (86.7%) patients, who did not respond to rituximab, successfully achieved remission after subsequently administration with obinutuzumab, which is comparable with patients without previous rituximab treatment. Studies from Lin et al. even reported that all (12/12, 100%) patients, who had previously received rituximab, achieved remission by obinutuzumab treatment (20). However, Su et al. demonstrated that only 64.3% of patients resistant to rituximab achieved remission after receiving obinutuzumab as an alternative therapy, which is significantly lower than the patients in the subgroup without prior rituximab treatment (64.3 vs. 91.1%) (23). Despite these varying results, the limited data also implied that obinutuzumab might have a promising efficacy for patients with unsatisfied response to rituximab. (**Supplementary Table S7**).

The efficacy of anti-CD20 antibody was mainly for its ability to induce immunological remission, including B cell depletion and auto-antibody clearance. For the patients who were positive for anti-PLA2R antibody, our study together with previous study had demonstrated that obinutuzumab could induce significant depletion of anti-PLA2R antibody, followed by clinical remission (15, 16, 23). However, for the patients with anti-PLA2R antibody negativity, whether obinutuzumab has the similar efficacy needs to be elucidated clearly. Patients with negative anti-PLA2R antibody in our study achieved a high response rate of 100.0% (16/16). Sethi S et al. also found that for the patients with anti-PLA2R antibody negativity, 100% (5/5) achieved clinical remission and up to 60% (3/5) achieved CR (17). These finding suggested that obinutuzumab had the same effect on the patients without circulating anti-PLA2R antibody. (**Supplementary Table S8**).

In our study, 38.2% of patients experienced AEs, most of which are tolerable infusion-related reactions and infections. While other studies have reported that obinutuzumab demonstrated a perfect safety profile with no serious adverse events among patients with pMN, our finding revealed that in some cases, obinutuzumab could lead to serious infections, even life-threatening complications. On one hand, the proportion of infections(23.6%), particularly in the alternative therapy group(10/40, 25.0%) in our study was slightly higher than the previous studies(8.5% (23)/16.7% (20)/19% (16)). On the other hand, though all the published studies reported no sever adverse events and deaths, 6 cases of severe pneumonia, particularly associated with COVID-19, occurred in our study. Among them, one patient ultimately died of respiratory failure, having experienced 8 rounds of immunomeds prior to the treatment of obinutuzumab. This highlights the need for caution when treating patients with a history of long-term immunosuppressive therapy, as there is an increased risk of severe infection that could lead to fatal outcomes.

Though all the patients were carefully screened for secondary factors and those with identifiable causes were excluded, the glomerular C1q and IgG1 positivity in our cohort were (8/35)22.9% and (19/22)86.4%. A cohort from China also reported glomerular C1q and IgG1 positivity in 22.9–23.2% and 97.6% of patients with pMN, respectively, which is consistent with our findings (25, 26). Using proximity ligation assay, Seifert et al. even detected C1q positivity in 34 of 39 patients and IgG1 positivity in 35 of 39 pMN patients (27). The discrepancies in glomerular C1q and IgG1 deposition across studies may be attributed to differences in detection methods used at various centers. Given the limited number of cases, the generalizability of these findings also remains to be further validated.

As a retrospective study, our study has several limitations. Firstly, the relatively small sample size and limited duration of follow-up, hinder generalizability and restrict further exploration of the effects of obinutuzumab on maintaining long-term remission and reducing relapse in pMN. Secondly, follow-up time points varied among patients, and not all clinical indicators were collected regularly. Therefore, we were unable to provide continuous trends at standardized post-treatment time points. This limits the ability to fully assess the dynamic changes over time. Thirdly, only 43/55(78.2%) patients underwent renal biopsy, while the remaining patients were diagnosed with pMN based on highly elevated level of anti-PLA2R antibody and the absence of secondary factors. Larger prospective studies are needed to confirm our findings.

In conclusion, our results suggest that obinutuzumab, used as both initial and alternative therapy, can induce a significant clinical response in patients with pMN, including those with impaired renal function, those with no response to rituximab and those with negative anti-PLA2R antibody. These results highlight obinutuzumab as a promising therapeutic option, expanding the range of strategies available for managing pMN.

## Data Availability

The raw data supporting the conclusions of this article will be made available by the authors, without undue reservation.
