# Supplementary material for: Efficacy and safety of obinutuzumab in primary membranous nephropathy: a real-world retrospective study
Source: Front Immunol. 2025 Aug 21;16:1650054. doi: 10.3389/fimmu.2025.1650054 (PMC12408494; doi:10.3389/fimmu.2025.1650054)
Supplement: Supplementary file 1 [file DataSheet1.docx]

**Supplementary Table S1. Characteristics at the time of diagnosis and previous therapies of patients with pMN in alternative therapy group.**

| Characteristics | N=40 |
| --- | --- |
| Positivity of anti-PLA2R antibody at baseline, n (%) | 39(97.5) |
| Level of anti-PLA2R antibody at baseline (RU/mL) | 99.1(39.8, 402.5) |
| Serum albumin at baseline (g/L) | 22.0(16.0, 29.0) |
| Proteinuria at baseline (g/24h) | 6.5(3.9, 12.0) |
| Serum creatinine at baseline (μmol/L) | 73.0(60.0, 84.0) |
| eGFR at baseline (mL/min/1.73 m^2^) | 92.9(81.3, 107.2) |
| Previous therapies |  |
| Rituximab, n (%) | 30(75.0) |
| Cyclophosphamide, n (%) | 9(22.5) |
| Calcineurin inhibitor, n (%) | 23(57.5) |
| Other immunosuppressants, n (%) | 10(25.0) |
| Rounds of immunomeds |  |
| 1 | 17(42.5) |
| 2 | 8(20.0) |
| 3 | 9(22.5) |
| 4 | 2(5.0) |
| 5 | 3(7.5) |
| >5 | 1(2.5) |
| Previous remission, n (%) | 6(15.0) |

MN: membranous nephropathy; eGFR: estimated glomerular filtration rate; PLA2R: M-type phospholipase A2 receptor.

**Supplementary Table S2. Pathological parameters of patients with pMN included in this study.**

|  | Alternative therapy  With renal biopsy  (N=32) | Initial therapy  With renal biopsy  (N=11) | *P*-value |
| --- | --- | --- | --- |
| Renal biopsy in our center, n (%) | 24(75) | 11(100) | 0.090 |
| MN stage |  |  |  |
| Ⅰ, n (%) | 2(8.3) | 0(0.0) | 1.000 |
| Ⅱ, n (%) | 14(58.3) | 3(27.3) | 0.146 |
| Ⅲ, n (%) | 8(33.3) | 8(72.7) | 0.065 |
| IgG deposition, n (%) | 24(100) | 11(100) | -- |
| IgG1 | 10/11(90.9) | 9/11(81.8) | 1.000 |
| IgG2 | 2/11(18.2) | 1/11(9.1) | 1.000 |
| IgG3 | 4/11(36.4) | 2/11(18.2) | 0.635 |
| IgG4 | 10/11(90.9) | 11/11(100) | 1.000 |
| IgA deposition, n (%) | 5(20.8) | 1(9.1) | 0.640 |
| IgM deposition, n (%) | 19(79.2) | 6(54.5) | 0.227 |
| C3 deposition, n (%) | 24(100) | 11(100) | -- |
| C1q deposition, n (%) | 6(25.0) | 2(18.2) | 1.000 |
| PLA2R staining, n (%) | 12/12(100.0) | 9(81.8) | 0.217 |
| Acute/subacute interstitial tubular injury, n (%) | 6(25.0) | 3(27.3) | 1.000 |

MN: membranous nephropathy; eGFR: estimated glomerular filtration rate; PLA2R: M-type phospholipase A2 receptor.

**Supplementary Table S3. Comparison of clinical characteristics and outcomes between patients with and without previous rituximab treatment.**

|  | With previous rituximab (N=30) | Without previous rituximab (N=10) | *P-*value |
| --- | --- | --- | --- |
| Rounds of immunomeds | 2(1.0, 3.0) | 1(1.0, 2.3) | 0.189 |
| Time to remission (months) | 10(6.8, 13.0) | 7.5(5.0, 10.0) | 0.209 |
| Clinical response, n (%) | 26(86.7) | 8(80.0) | 0.629 |
| Partial remission, n (%) | 12(40.0) | 6(60.0) | 0.300 |
| Complete remission, n (%) | 14(46.7) | 2(20.0) | 0.263 |
| Relapse, n (%) | 2/26(7.7) | 0(0.0) | 1.000 |

**Supplementary Table S4. Comparison of clinical outcomes between anti-PLA2R antibody positive and negative subgroups in patients with pMN receiving obinutuzumab treatment**

|  | Anti-PLA2R antibody negativity  (N=16) | Anti-PLA2R antibody positivity  (N=39) | *P-*  value |
| --- | --- | --- | --- |
| Clinical response, n (%) | 16(100.0) | 30(76.9) | **0.046** |
| Complete remission, n (%) | 7(43.8) | 17(43.6) | 1.000 |

**Supplementary Table S5. Comparison of clinical characteristics and outcomes of pMN patients with eGFR<60 mL/min/1.73m^2^ from our cohort and cases published in other studies at administration of obinutuzumab.**

|  | Our cohort  (N=18) | References^&^  (N=15) | *P*  value |
| --- | --- | --- | --- |
| Gender (M/F), n | 9/9 | 9/6 | 0.729 |
| Age (years) | 61.9±9.5 | 57.7±13.5 |  |
| Previous rituximab, n (%) | 14(77.8) | 12/14(85,7) | 0.672 |
| Previous calcineurin inhibitor, n (%) | 9(50.0) | 8/14(57.1) | 0.735 |
| Previous cyclophosphamide, n (%) | 6(33.3) | 5/14(35.7) | 1.000 |
| eGFR at administration (mL/min/1.73 m^2^) | 35.3(27.8, 52.9) | 42.0(34.0, 52.6) | 0.057 |
| eGFR at last visit (mL/min/1.73 m^2^) | 47.6(43.3, 69.2)* | 51.1(45.7, 57.6) | 0.909 |
| Proteinuria at administration (g/24h) | 4.1(3.2, 12.5) | 9.7(5.8, 14.3) | 0.124 |
| Proteinuria at last visit (g/24h) | 1.1(0.2, 4.1)** | 2.1(1.2, 5.9)^###^ | 0.073 |
| Serum albumin at administration (g/L) | 24.0(21.8, 32.0) | 26.2(21.0, 28.0) | 0.365 |
| Serum albumin at last visit (g/L) | 40.0(37.0, 42.0)*** | 38.0(32.0, 39.9)^###^ | 0.101 |
| Level of anti-PLA2R antibody at administration (RU/mL) | 48.1(5.0, 138.1) | 100.7(62.5, 181.8) | 0.686 |
| Level of anti-PLA2R antibody at last visit (RU/mL) | 5.0(5.0, 5.0)*** | 2.3(1.7, 5.0)^###^ | **0.029** |
| Follow-up duration (months) | 12.0(8.0, 15.3) | 10(6, 18) |  |
| Clinical response, n (%) | 13(72.2) | 10(66.7) | 1.000 |
| Complete remission, n (%) | 6(33.3) | 2(13.3) | 0.242 |

eGFR: estimated glomerular filtration rate; PLA2R: M-type anti-Phospholipase A2 receptor antibody. ^*^*P* < 0.05, ^**^*P* < 0.01 and ^***^*P* < 0.001 comparison before and after obinutuzumab treatment in our cohort; ^#^*P* < 0.05, ^##^*P* < 0.01 and ^###^*P* < 0.001 comparison before and after obinutuzumab treatment in references.

&: Date were pooled from previous case reports that included patients with eGFR < 60mL/min/1.73m^2^ at the initiation of obinutuzumab treatment. 15 patients were collected (3 patients from Klomjit N et al. [1]; 2 patients from Naik S et al. [2]; 7 patients from Sethi S et al. [3]; 1 patient from Hao J et al. [4]; 1 patient from Zhang Y et al. [5]; 1 patient from Francisco J et al. [6])

**Supplementary Table S6. Clinical outcomes in our cohort and other studies for pMN patients receiving obinutuzumab treatment as initial therapy or alternative therapy.**

| References | Therapy | No. of patients | Clinical  response | CR | Immunological remission |
| --- | --- | --- | --- | --- | --- |
| Su X et al. [7] | Initial therapy | 20 | 18(90.0%) | 7(35.0%) | 16(94.1%) |
|  | Second-line therapy | 39 | 32(82.0%) | 13(33.3%) | 27(87.1%) |
| Xu M et al. [8] | Refractory | 20 | 9/10(90.0%) | 2/10(20%) | -- |
| Hao J et al. [9] | Untreated | 12 | 10(83.3%) | 2(16.7%) | 6/6(100.0%) |
| Hu X et al. [10] | Untreated | 21 | 20(95.0%) | 8(38.0%) | -- |
| Our corhort | Alternative therapy | 40 | 34(85.0%) | 16(40.0%) | 15/16(93.8%) |
|  | Initial therapy | 15 | 12(80.0%) | 8(53.3%) | 8/9(88.9%) |

**Supplementary Table S7. Efficiency of obinutuzumab on clinical outcomes in our cohort and other studies for pMN patients with or without previous rituximab treatment.**

| References | Previous RTX | No. of patients | Clinical response | CR |
| --- | --- | --- | --- | --- |
| Su X et al. [7] | With | 14 | 9(64.3) | 3(21.4) |
|  | Without | 45 | 41(91.1) | 17(37.8) |
| Lin Y et al. [11] | With | 12 | 12(100.0) | 4(33.3) |
|  | Without | 6 | 5(83.3) | 1(16.7) |
| Our cohort | With | 30 | 26(86.7) | 14(46.7) |
|  | Without | 10 | 8(80.0) | 2(20.0) |

**Supplementary Table S8. Efficiency of obinutuzumab on clinical outcomes in our cohort and another study for pMN patients with undetectable anti-PLA2R antibody in circulation**

| References | No. of patients | Clinical response | CR |
| --- | --- | --- | --- |
| Sethi S et al. [3] | 5 | 5(100.0%) | 3(60.0%) |
| Our cohort | 16 | 16(100.0%) | 7(43.8%) |

**Supplementary Figure legend**

**Supplementary Figure S1. Kaplan-Meier curves for the cumulative incidence of clinical remission in patients with and without previous rituximab treatment before receiving obinutuzumab treatment.**

RTX: rituximab.

**Supplementary Figure S1**


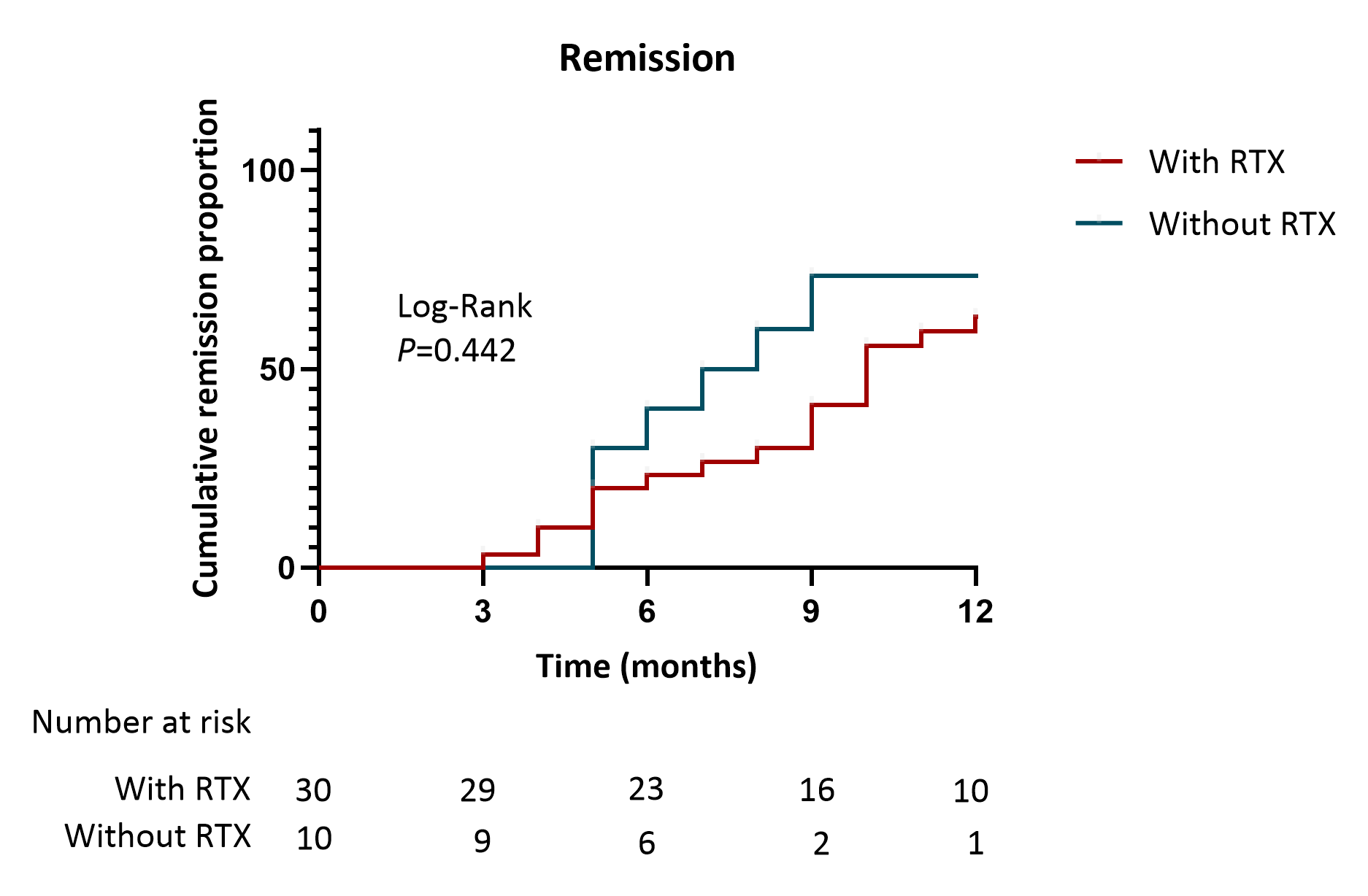


**Supplementary References**

1. Klomjit N, Fervenza FC, Zand L (2020) Successful treatment of patients with refractory pla(2)r-associated membranous nephropathy with obinutuzumab: a report of 3 cases. Am J Kidney Dis 76(6):883-888. <https://doi.org/10.1053/j.ajkd.2020.02.444>
2. Naik S, Shukla S, Av N, Kumar V, Sekar A, Nada R, Rathi M, Kohli HS, Ramachandran R (2023) Obinutuzumab in refractory phospholipase a2 receptor-associated membranous nephropathy with severe CKD. Kidney Int Rep 8(4):942-943. https://doi.org/10.1016/j.ekir.2023.01.035
3. Sethi S, Kumar S, Lim K, Jordan SC (2020) Obinutuzumab is effective for the treatment of refractory membranous nephropathy. Kidney Int Rep 5(9):1515-1518. https://doi.org/10.1016/j.ekir.2020.06.030
4. Hao J, Wang J, Zhou P, Xu R, Chen X (2024) Obinutuzumab in untreated primary membranous nephropathy: an observational case series. Nephrology (Carlton) 29(11):709-716. https://doi.org/10.1111/nep.14331
5. Zhang Y, Sun J, Gao J, Sun W, Xu L, Zhai C, SuW and Wang H (2024) Case report: one case of refractory membranous nephropathy with hypokalemia after rituximab infusion was switched to obinutuzumab without recurrence of hypokalemia. Front Pharmacol. 15:1347880. <https://doi.org/10.3389/fphar.2024.1347880>
6. Francisco J, Melissa CC, Marina AT, Javier BM, Fabiola AG, Mercedes SL (2024) Obinutuzumab in the treatment of PLA2R-positive membranous glomerulonephritis resistant to treatment. Nephrology 44(2):306-307. https://doi.org/10.1016/j.nefroe.2024.03.004.
7. Su X, Wu B, Tie X, Guo X, Feng R, Qiao X, Wang L (2024) Obinutuzumab as initial or second-line therapy in patients with primary membranous nephropathy. Kidney Int Rep 9(8):2386-2398. https://doi.org/10.1016/j.ekir.2024.05.004
8. Xu M, Wang Y, Wu M, Chen R, Zhao W, Li M, Hao CM, Xie Q (2024) Obinutuzumab versus rituximab for the treatment of refractory primary membranous nephropathy. Nephrol Dial Transplant 0(0). <https://doi.org/10.1093/ndt/gfae230>
9. Hao J, Wang J, Zhou P, Xu R, Chen X (2024) Obinutuzumab in untreated primary membranous nephropathy: an observational case series. Nephrology (Carlton) 29(11):709-716. <https://doi.org/10.1111/nep.14331>
10. Hu X, Zhang M, Xu J, Gao C, Yu X, Li X, Ren H, Wang W, Xie J (2024) Comparison of obinutuzumab and rituximab for treating primary membranous nephropathy. Clin J Am Soc Nephrol 19(12):1594-1602. <https://doi.org/10.2215/CJN.0000000000000555>
11. Lin Y, Han Q, Chen L, Wang Y, Ren P, Liu G, Lan L, Lei X, Chen J, Han F (2024) Obinutuzumab in refractory membranous nephropathy: a case series. Kidney Med 6(8):100853. https://doi.org/10.1016/j.xkme.2024.100853
